# Using a cross-cohort comparison design to test the role of maternal smoking in pregnancy in child mental health and learning: evidence from two UK cohorts born four decades apart

**DOI:** 10.1093/ije/dyaa001

**Published:** 2020-02-10

**Authors:** Ruth Sellers, Naomi Warne, Frances Rice, Kate Langley, Barbara Maughan, Andrew Pickles, Anita Thapar, Stephan Collishaw

**Affiliations:** d1 Rudd Centre for Adoption Research and Practice, School of Psychology, University of Sussex, Brighton, UK; d2 Division of Psychological Medicine and Clinical Neurosciences, Cardiff University School of Medicine, Child and Adolescent Psychiatry, MRC Centre for Neuropsychiatric Genetics and Genomics, Cardiff, Wales; d3 Cardiff University, School of Psychology, Cardiff, Wales; d4 Social, Genetic and Developmental Psychiatry Centre, Institute of Psychiatry, Psychology and Neuroscience, King’s College London, London, UK; d5 Department of Biostatistics and Health Informatics, Institute of Psychiatry, Psychology and Neuroscience, King’s College London, London, UK

**Keywords:** Maternal smoking, hyperactivity, conduct problems, cognitive, birth weight, causal inference, triangulation, cross-cohort comparison design

## Abstract

**Background:**

Maternal smoking in pregnancy is associated with low birth weight (LBW), child conduct problems, hyperactivity and lower cognitive attainment, but associations may reflect measured and unmeasured confounding. Cross-cohort designs can aid causal inference through comparison of associations across populations with different confounding structures. We compared associations between maternal smoking in pregnancy and child conduct and hyperactivity problems, cognition and LBW across two cohorts born four decades apart.

**Methods:**

Two national UK cohorts born in 1958 (*n* = 12 415) and 2000/01 (*n* = 11 800) were compared. Maternal smoking in pregnancy and child birth weight was assessed at or shortly after birth. Parents rated children’s conduct problems and hyperactivity, and children completed standardized tests of reading and mathematics.

**Results:**

Maternal smoking in pregnancy was less common and more strongly associated with social disadvantage in 2000/01 compared with 1958 (interactions *P* < 0.001). Maternal smoking in pregnancy was robustly and equivalently associated with infant LBW in both cohorts [interactions: boys odds ratio (OR) = 1.01 (0.89, 1.16), *P* = 0.838; girls OR = 1.01 (0.91, 1.17), *P* = 0.633]. Maternal smoking was more strongly associated with conduct problems, hyperactivity and reading in the 2000/01 cohort (interactions *P* < 0.001).

**Conclusions:**

Marked cross-cohort change in associations between maternal smoking and child conduct problems, hyperactivity and reading highlights the likely role of confounding factors. In contrast, association with LBW was unaffected by change in prevalence of maternal smoking and patterns of confounding. The study highlights the utility of cross-cohort designs in helping triangulate conclusions about the role of putative causal risk factors in observational epidemiology.

**Key Messages**

- Comparison of associations across populations with different rates of risk factors and different confounding structures can aid causal inference about the relationship between putative risk exposures and outcomes.
- The study used a cross-cohort comparison design to test the relationship between maternal smoking in pregnancy and children’s outcomes using data from two cohorts born four decades apart.
- Maternal smoking was less prevalent and more strongly associated with social disadvantage in the more recent cohort.
- Evidence is consistent with a causal effect of maternal smoking on child birth weight, but links between maternal smoking and child conduct problems, hyperactivity and reading are likely influenced by confounding.

## Introduction

Maternal smoking in pregnancy shows robust statistical association with child Attention Deficit Hyperactivity Disorder (ADHD) and conduct problems in observational studies.^1–3^ However, as recently reviewed,^4^ genetically sensitive designs involving siblings who are discordant for exposure,^5–7^ children born by assisted conception^8^^,^^9^ and ‘negative control exposure’ designs (e.g. comparing maternal and paternal smoking)^10^ demonstrate that the association is likely to be explained by unmeasured genetic and/or social confounders. Maternal smoking in pregnancy has also been associated with children’s cognitive ability, but studies again suggest that these associations may be explained by confounding factors such as social disadvantage and maternal education.^11–14^ In contrast, studies using a variety of designs point to maternal smoking in pregnancy having causal biologically-mediated effects on intra-uterine growth and birth weight.^4^^,^^8^^,^^10^^,^^14^^,^^15^

One explanation for these findings is that person–environment correlation accounts for observed associations between maternal smoking and children’s developmental outcomes whereby maternal characteristics and family adversity are associated both with maternal smoking in pregnancy and with children’s outcomes.^16^ For example, mothers living in more deprived circumstances and those with increased vulnerability for psychiatric illness are more likely to smoke in pregnancy,^10^^,^^17^ and there are well-established links between family adversity, parental psychopathology and children’s behavioural and cognitive outcomes.^18–20^ Standard methods of covariate adjustment are helpful in reducing biases though they require a priori knowledge of what relevant confounders to include, and confounders will be measured imperfectly. Standard methods can also not rule out residual unmeasured confounding.^4^^,^^21^^,^^22^ Genetically informed designs and negative control studies are important for addressing this problem, but each come with particular assumptions and potential limitations. Triangulation using a range of alternative approaches is important in drawing robust conclusions.^23^

An additional method for aiding causal inference is the cross-cohort comparison design which utilizes between-population differences in patterns of confounding.^24^ The logic here is that the strength of an association between an exposure and outcomes should be independent of the prevalence and social correlates of the exposure variable in the case of a true causal effect, but will vary substantially between cohorts with different confounding structures if associations are largely explained by confounding.

The current study focuses on maternal smoking in pregnancy, which in one previous study using an international cross-cohort design was considered as a potential risk exposure for child conduct problems, hyperactivity and emotional problems.^25^ The study found evidence consistent with a causal risk association for child conduct problems but not hyperactivity or emotional problems. We use an analogous cross-cohort study design based on historical change in societal rates and correlates of maternal smoking. Over recent decades, there have been major changes in public understanding of the negative consequences of smoking in general, and specifically during pregnancy. Fewer women now smoke in pregnancy, and those who do are more likely to come from socially deprived backgrounds.^17^^,^^26^ The current study capitalizes on these temporal trends in patterns of confounding to extend the cross-cohort comparison design by examining two large UK national cohorts born 40 years apart.

We aimed to test for cross-cohort change in associations between maternal smoking in pregnancy and children’s developmental outcomes: low birth weight (LBW), conduct problems, hyperactivity and cognitive attainment (reading and mathematics). We hypothesized that maternal smoking would show equivalent associations with infant LBW (consistent with a causal effect), but would show stronger associations with conduct problems, hyperactivity and cognitive outcomes in the more recent cohort (implicating confounding).

### Methods

#### Samples and design

Two UK population cohorts were compared. The National Child Development Study (NCDS) is a longitudinal birth cohort of children born in one week (3–9 March) in 1958 in England, Wales and Scotland.^27^ Pre- and peri-natal factors were assessed at birth, and follow-up data was available at age 7 years. Analyses were conducted with 12 415 families (49% girls) with data available at birth and age 7 years. The Millennium Cohort Study (MCS) is a longitudinal birth cohort of children born between September 2000 and January 2002 in England, Wales, Scotland and Northern Ireland.^28^ Pre-and peri-natal information was collected when the child was 9 months old, with follow-up data available at age 7 years. Analyses included 11 800 families (51% girls) with available data on predictors and outcome variables.

#### Measures

##### Perinatal factors

Pre- and peri-natal factors were assessed in NCDS (1958) at birth using midwife reports, and in MCS (2000) at 9 months by maternal reports. Both cohorts included information on maternal smoking in pregnancy (yes/no) and infant birth weight (coded as LBW if <2500 g). Previous work (in other samples) has shown very strong agreement between antenatal records and later maternal reports of birth weight (*r* = 0.99) and of smoking in pregnancy and LBW (kappas >0.8).^29^

##### Child conduct problems and hyperactivity (age 7 years)

Child conduct and hyperactivity problems were assessed in MCS using the parent version of the Strengths and Difficulties Questionnaire (SDQ). The SDQ is a well-validated symptom screen with individual symptoms rated as 0 ‘does not apply’, 1 ‘applies somewhat’ and 2 ‘certainly applies’.^30^ Mean conduct problem and hyperactivity scores ranged from 0 to 10 and were utilized as the main outcome measures. For descriptive purposes and supplementary analyses, we identified children scoring in the abnormal range for each subscale using SDQ cut-points.^30^^,^^31^ The NCDS used the precursor to the SDQ, the parent-completed Rutter-A scale^32^^,^^33^ which included 11 closely comparable items. To ensure consistency of measurement of conduct problems and hyperactivity across the two cohorts, independent calibration data (where parents completed both measures) were used to impute SDQ-equivalent conduct problem and hyperactivity scores for NCDS.^34^ To reflect uncertainty in the calibrated values we used multiple imputation^35^ with 20 imputed datasets. Confidence intervals (CIs) for statistical estimates thus reflect both between-individual variation in scores, as well as imprecision of calibration as reflected by variation in scores across the multiply imputed datasets.

##### Cognitive outcomes (age 7 years)

Child reading and mathematics tests were completed by children at school in NCDS (1958 birth cohort) and administered by interviewers in home-based assessments in MCS (2000/01 cohort). NCDS used the Southgate Reading test, a 31-item word recognition test (α = 0.95) and a ten-item arithmetic test designed by the National Foundation for Educational Research (α = 0.92).^36^ The MCS cohort used the British Ability Scale Word Reading assessment (α = 0.93) and an adapted version of the National Foundation for Educational Research Progress in Maths test (α = 0.98).^37^ Test scores were standardized within each cohort.

##### Measures of family adversity (age 7 years)

Several measured potential confounders were included: family housing tenure [rented vs ‘homeowner’ (owned outright or with mortgage)], parental marital status (unmarried vs married), maternal education beyond statutory minimum leaving age (no vs yes), occupational status (manual vs non-manual) and maternal age at birth.

#### Non-response

Response rates at 7 years were 86.7 and 72.0% for the earlier and later cohorts respectively. Inverse probability response weights were estimated for NCDS using predicted values derived from logistic regression analyses of predictors of non-response.^34^ Standard analytic procedures and sampling weights, developed for use with MCS, were employed to account for patterns of non-response and also to correct for the stratified cluster sample design.^38^

#### Analysis strategy

Initial analyses compared rates of maternal smoking in pregnancy for the two cohorts, as well as associations between maternal smoking and indicators of family adversity. Interactions by cohort tested whether maternal smoking had become more strongly associated with family adversity over time.

To test primary study aims, we examined associations between maternal smoking in pregnancy and each of the child outcomes: infant LBW and conduct problems, hyperactivity, reading and mathematics at age 7 years. Tests of interactions (between cohort and maternal smoking) assessed whether associations with each child outcome differed over time. Analyses were conducted separately for boys and girls. Secondary analyses adjusted for sociodemographic factors to examine whether this helped to explain any differences in associations. All analyses (conducted in Stata version 13.1^39^) included the survey command and sample-specific weights to account for survey design and sample attrition, and using the MIM command^40^ which combines parameter estimates from across the 20 imputed datasets. The reported results reflect both within-dataset variation in parameter estimates [standard errors (SEs)] and between-dataset variation in parameter estimates (calibration uncertainty), thus representing a conservative approach.^34^

## Results

### Cross-cohort comparison of maternal smoking in pregnancy: changes in prevalence and associations with family adversity

**Figure 1.**
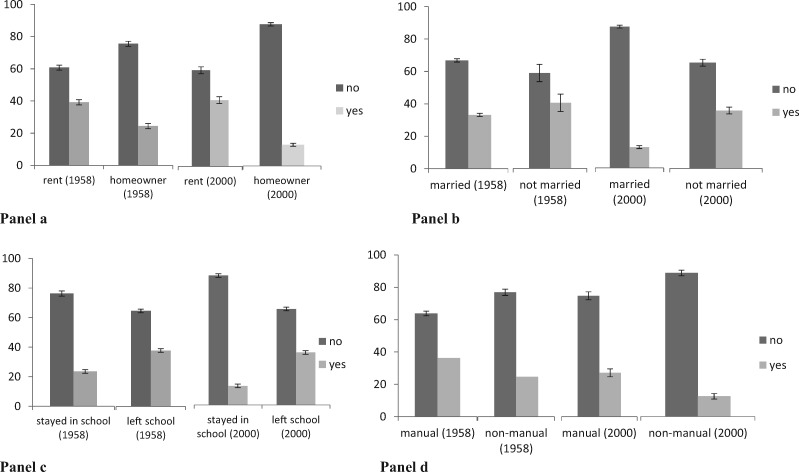
Percentage of those who smoked or did not smoke in pregnancy by family adversity for each cohort. Panel a: percentage smoking in pregnancy by housing tenure by cohort. Panel b: percentage smoking in pregnancy by marital status at birth of child by cohort. Panel c: percentage smoking in pregnancy by education status by cohort. Panel d: percentage smoking in pregnancy by occupational status by cohort. Home ownership includes owned outright or with mortgage.

As expected, fewer mothers had smoked in pregnancy in MCS (2000/01: 22%) compared with NCDS [1958: 33%; OR = 0.79 (0.77, 0.81), *P* < 0.001]. Patterns of confounding also differed between the two cohorts. Maternal smoking in pregnancy was more strongly associated with measures of family adversity in the later cohort, MCS (Fig. 1 and Table 1).

**Table 1.** Cross-cohort comparison of associations between maternal smoking in pregnancy and family adversity; home ownership includes owned outright or with mortgage

	Association of maternal smoking in pregnancy (MSIP) and family adversity by cohort		
	1958 Cohort (NCDS)	2000/01 Cohort (MCS)	Cohort x MSIP interaction
	OR/b (95% CI), *P*	OR/b (95% CI), *P*	OR/b (95% CI), *P*
Home ownership	0.51 (0.45, 0.56), *P* < 0.001	0.22 (0.19, 0.25), *P* < 0.001	0.66 (0.61, 0.71), *P* < 0.001
Married at birth	0.74 (0.58, 0.93), *P* < 0.05	0.27 (0.24, 0.30), *P* < 0.001	0.61 (0.54, 0.69), *P* < 0.001
Low maternal education	1.90 (1.70, 2.13), *P* < 0.001	3.72 (3.34, 4.14), *P* < 0.001	1.41 (1.30, 1.52), *P* < 0.001
Occupational status (non-manual)	0.56 (0.49, 0.63), *P* < 0.001	0.39 (0.33, 0.46), *P* < 0.001	0.83 (0.76, 0.92), *P* < 0.001
Maternal age at birth, years	0.13 (−0.13, 0.40), *P* = 0.439	−2.60 (−2.88, −2.31), *P* < 0.001	−2.73 (−3.12, −2.35), *P* < 0.001

### Cross-cohort comparison of infant LBW, child conduct problems and hyperactivity

As shown in Table 2, there was no change in the proportion of children born with LBW from 1958 (NCDS) to 2000 (MCS) for boys [odds ratio (OR) = 1.03 (0.96, 1.09), *P* = 0.439] or girls [OR = 0.99 (0.93, 1.04), *P* = 0.617].

**Table 2.** Prevalence of low birth weight, conduct, hyperactivity, reading and mathematics problems by cohort and gender

	Boys		
	1958 Cohort (NCDS) *n* = 6344	2000/01 Cohort (MCS) *n* = 5731	b/OR (95% CI), *P*
Low birth weight, % (95% CI)	5.7 (5.2, 6.2)	6.0 (5.5, 6.5)	1.03 (0.96, 1.09), *P =* 0.439
Conduct, mean (SE)	1.70 (0.19)	1.59 (0.03)	−0.11 (−0.48, 0.26), *P =* 0.579
Hyperactivity, mean (SE)	3.47 (0.18)	3.87 (0.04)	0.40 (0.01, 0.80), *P* = 0.046
Conduct, % high (95% CI)	12.6 (6.8, 18.4)	12.9 (11.9, 13.9)	1.02 (0.79, 1.31), *P* = 0.850
Hyperactivity, % high (95% CI)	14.2 (7.9, 20.5)	17.2 (16.1, 18.4)	1.13 (0.87, 1.47), *P* = 0.343
Reading, < −1SD^a^, % (95% CI)	16.6 (15.7, 17.6)	27.1 (25.4, 28.7)	1.37 (1.29, 1.44), *P* < 0.001
Maths, < −1SD^a^, % (95% CI)	20.7 (19.7, 21.8)	21.0 (19.5, 22.5)	1.01 (0.95, 1.07), *P* = 0.758

	Girls		

	*n* = 6071	*n* = 6304	

Low birth weight, % (95% CI)	7.3 (6.7, 7.8)	7.1 (6.5, 7.6)	0.99 (0.93, 1.04), *P* = 0.617
Conduct, mean (SE)	1.52 (0.12)	1.27 (0.02)	−0.25 (−0.48, −0.02), *P* = 0.026
Hyperactivity, mean (SE)	2.69 (0.18)	2.95 (0.04)	0.26 (−0.08, 0.62), *P* = 0.143
Conduct, % high (95% CI)	14.7 (11.1, 18.7)	8.1 (7.2, 8.9)	0.71 (0.63, 0.82), *P* < 0.001
Hyperactivity, % high (95% CI)	10.8 (3.2, 18.5)	9.2 (8.4, 10.0)	0.94 (0.65, 1.35), *P* = 0.725
Reading, < −1SD^a^, % (95% CI)	17.8 (16.8, 18.8)	17.2 (15.7, 18.6)	0.98 (0.92, 1.04), *P* = 0.435
Maths, < −1SD^a^, % (95% CI)	19.5 (18.5, 20.6)	19.0 (17.6, 20.5)	0.98 (0.93, 1.04), *P* = 0.586

a Standardized within each cohort. Note, values of *n* are based on weighted proportions where data is available on all key outcomes (conduct, hyperactivity, reading and maths problems).

There was a small increase in mean hyperactivity problems for boys [b = 0.40 (0.01, 0.80), *P* = 0.046]. There was a decrease in mean conduct problems for girls [b = −0.25 (−0.48, −0.02), *P* = 0.026] and a reduction in the proportion of girls scoring in the abnormal range for conduct problems [OR = 0.71, (0.61, 0.82), *P* < 0.001].

### Maternal smoking in pregnancy and infant LBW

As hypothesized, maternal smoking in pregnancy was strongly and equivalently associated with LBW in both cohorts (see Table 3). Tests of interaction found no difference in effect size for boys [cohort x smoking interaction: OR = 1.01 (0.89, 1.16), *P* = 0.838] or for girls [cohort interaction: OR = 1.01 (0.91, 1.17), *P* = 0.633].

**Table 3.** Cross-cohort comparison of associations between maternal smoking in pregnancy (MSIP) and child outcomes

		1958 Cohort (NCDS)			2000/01 Cohort (MCS)			Cohort x MSIP interaction
	Child outcome	Maternal smoking	No maternal smoking	OR/b (95% CI), *P*	Maternal smoking	No maternal smoking	OR/b (95% CI), *P*	OR/b (95% CI), *P*
Boys	LBW, %	7.7%	4.7%	1.72 (1.42, 2.07), *P* < 0.001	8.7%	5.1%	1.76 (1.47, 2.12), *P* < 0.001	1.01 (0.89, 1.16), P = 0.838
	Conduct, mean (SE)	1.85 (0.22)	1.62 (0.18)	0.23 (0.07, 0.41), *P* < 0.009	2.19 (0.06)	1.39 (0.03)	0.80 (0.66, 0.94), *P* < 0.001	0.57 (0.44, 0.70), *P* < 0.001
	Hyperactivity, mean (SE)	3.63 (0.20)	3.38 (0.19)	0.25 (0.06, 0.43), *P* < 0.010	4.70 (0.08)	3.59 (0.04)	1.11 (0.94, 1.28) *P* < 0.001	0.86 (0.76, 0.98), *P* < 0.001
	Reading, mean (SE)	−0.19 (0.02)	0.09 (0.02)	−0.28 (−0.34, −0.22), *P* < 0.001	−0.49 (0.04)	−0.04 (0.03)	−0.45 (−0.55, −0.35), *P* < 0.001	−0.17 (−0.28, −0.06), *P* < 0.003
	Maths, mean (SE)	−0.20 (0.02)	0.12 (0.02)	−0.32 (−0.37, −.26), *P* < 0.001	−0.25 (0.04)	0.10 (0.03)	−0.35 (−0.45, −0.25), *P* < 0.001	−0.03 (−0.14, 0.08), *P* = 0.545
Girls	LBW, %	10.0%	5.8%	1.83, (1.54, 2.17), *P* < 0.001	10.9%	5.9%	1.94 (1.63, 2.31), *P* < 0.001	1.03 (0.91, 1.17), *P* = 0.633
	Conduct, mean (SE)	1.63 (0.13)	1.47 (0.11)	0.16 (0.03, 0.28), *P* < 0.013	1.71 (0.05)	1.14 (0.03)	0.56 (0.45, 0.67), *P* < 0.001	0.40 (0.32, 0.48), *P* < 0.001
	Hyperactivity, mean (SE)	2.85 (0.20)	2.62 (0.18 )	0.23 (0.05, 0.41), *P* < 0.011	3.62 (0.08)	2.77 (0.04)	0.85 (0.68, 1.02), *P* < 0.001	0.62 (0.52, 0.72), *P* < 0.001
	Reading, mean (SE)	−0.14 (0.02)	0.09 (0.02)	−0.23 (−0.28, −0.18), *P* < 0.001	−0.18 (0.04)	0.21 (0.02)	−0.38 (−0.47, −0.30), *P* < 0.001	−0.15 (−0.26, −0.04), *P* < 0.004
	Maths, mean (SE)	−0.21 (0.02)	0.08 (0.02)	−0.28 (−0.34, −0.23), *P* < 0.001	−0.25 (0.04)	0.03 (0.02)	−0.28 (−0.36, −0.20), *P* < 0.001	0.00 (−0.10, 0.10), *P* = 0.953

### Maternal smoking in pregnancy and child conduct problems and hyperactivity

For boys and girls in both cohorts, there were associations between maternal smoking in pregnancy and child conduct and hyperactivity problems at age 7 years (see Table 3). However, relative to the earlier cohort (NCDS), maternal smoking in pregnancy in the later cohort (MCS) was more strongly associated with child conduct problems [cohort x maternal smoking interactions: boys b = 0.57 (0.44, 0.70), *P* < 0.001; girls b = 0.40 (0.32, 0.48), *P* < 0.001] and child hyperactivity [interactions: boys b = 0.86 (0.76, 0.98), *P* < 0.001; girls b = 0.62 (0.52, 0.72), *P* < 0.001]. Sensitivity analyses focusing on abnormal range scores showed equivalent results (Supplementary Table 1, available as Supplementary data at *IJE* online).

### Maternal smoking in pregnancy and child cognitive attainment

Maternal smoking in pregnancy was associated with child mean standardized reading and mathematics attainment in both cohorts (Table 3). Maternal smoking in pregnancy became more strongly associated with child reading attainment in the later cohort [cohort x maternal smoking interactions: boys β=−0.17 (−0.28, −0.06), *P* = 0.003; girls β = −0.15 (−0.26, −0.04), *P* = 0.004]. In contrast, associations between maternal smoking in pregnancy and mathematics scores did not differ between the two cohorts [interactions: boys β=−0.03 (−0.14, 0.08), *P* = 0.545; girls β = 0.00 (−0.10, 0.10), *P* = 0.953]. Sensitivity analyses focusing on the categorically defined reading and maths problems (< −1SD vs > −1SD) showed equivalent results (Supplementary Table 2, available as Supplementary data at *IJE* online).

### Secondary analyses: explaining differences in patterns of association

Additional analyses tested the extent to which differences in associations between maternal smoking during pregnancy and child conduct problems, hyperactivity and reading were attenuated when adjusting for measured sociodemographic covariates. Estimates of the cohort x smoking interaction terms were attenuated in each case—conduct problems [boys: unadjusted b = 0.57 [0.44, 0.70], *P* < 0.001; adjusted b = 0.34 (0.22, 0.48), *P*< 0.001; girls: unadjusted b = 0.40 (0.32, 0.48), *P* < 0.001; adjusted b = 0.20 (0.12, 0.28), *P* < 0.001]; hyperactivity [boys: unadjusted b = 0.86 (0.76, 0.98) *P* < 0.001; adjusted b = 0.58 (0.46, 0.70), *P* < 0.001; girls: unadjusted b = 0.62 (0.52, 0.72), *P* < 0.001; adjusted b = 0.28 (0.18, 0.38), *P* < 0.001); reading [boys: unadjusted b = −0.17 (-0.28, −0.06), *P* = 0.003; adjusted b = 0.05 (−0.04, 0.12), *P* = 0.325; girls: unadjusted b = −0.15 (−0.26, −0.04), *P* = 0.004; adjusted b = 0.03 (−0.03, 0.10), *P* = 0.349].

## Discussion

Maternal smoking in pregnancy remains common globally, despite a historical drop in population prevalence.^17^^,^^41^ It is targeted with some success in public health interventions including health information campaigns and via smoking-cessation support. Nevertheless, as many as half of women smokers continue to smoke during pregnancy.^42^ There is established evidence that this causes harm to mothers’ own health and presents a risk to a healthy pregnancy and the developing foetus.^43^ There has also been speculation that maternal smoking in pregnancy might also be linked to a wider range of children’s later developmental outcomes including children’s behaviour, neurodevelopmental problems and cognitive attainment. However, understanding the causal nature of observed associations is important, because if there is evidence of causation, then this would mean that reducing maternal smoking in pregnancy should be a prime target for public health interventions aimed at improving these child outcomes. If not, then it is important to acknowledge that successfully reducing maternal smoking rates, though important for other reasons, will likely not result in improvements in these associated outcomes. Instead, greater priority should be given to identifying alternative, causal avenues for improving children’s mental health, behaviour and cognitive development.

Testing causal effects using observational study designs is challenging, but a variety of different designs that approximate natural experiments do exist (e.g. children of twin studies; children born by IVF). These study designs have led to important insights into whether exposures such as maternal smoking in pregnancy have causal effects on child development, demonstrating that many observed exposure–outcome associations (e.g. maternal smoking in pregnancy and child ADHD) likely reflect the influence of common confounders. However many of these designs utilize relatively rare population subgroups and this potentially affects the power to detect small effects and the generalizability of study findings. It is therefore important to triangulate evidence with whole-population study designs.^4^^,^^22^^,^^23^

The current study aimed to extend evidence regarding associations between maternal smoking in pregnancy and children’s developmental outcomes using a cross-cohort comparison design. We compared associations across two UK national cohort studies born 40-years apart that showed major differences in the prevalence and patterning of social confounders of maternal smoking in pregnancy.

Prior studies using genetically sensitive designs provide evidence of a likely causal link between maternal smoking in pregnancy and infant LBW.^9^^,^^10^^,^^14^^,^^15^ The findings of the present study are consistent with this, showing a robust and equivalent association between maternal smoking in pregnancy and LBW in both cohorts. These results are as we hypothesized, and therefore provide proof of principle that the cross-cohort comparison design works as expected and is a useful complement to other research designs testing causal explanations.

In contrast, maternal smoking in pregnancy was substantially more strongly associated with child hyperactivity and conduct problems in the later cohort which as we have shown was much more strongly affected by confounding with family adversity. Previous literature has suggested that the association between maternal smoking in pregnancy and child mental health may not be causal^4^ and that maternal smoking in pregnancy is a marker of a wide range of confounding factors such as maternal depression, social disadvantage and inherited factors that themselves are associated with negative child outcomes.^4^^,^^14^^,^^17^ In the period considered in this cross-cohort comparison study, rates of maternal smoking in pregnancy reduced and associations with observed measures of family adversity increased. Increased associations between maternal smoking in pregnancy and child conduct problems and hyperactivity are therefore likely explained by cross-cohort differences in confounding.

A similar pattern of findings was evident for analyses of children’s reading attainment. Maternal smoking during pregnancy was associated with reading in both cohorts. However, this association was stronger in the later cohort. This provides support, along with evidence from other designs,^11–14^ that the association between maternal smoking in pregnancy and children’s reading development is at least in part attributable to confounding variables. Findings suggested some differences in findings for children’s mathematics attainment with evidence for equivalent associations with maternal smoking in pregnancy in the two cohorts. Previous research has suggested that along with strong commonalities in the development of reading and maths there are also important differences in their neurocognitive underpinnings.^44^ However, caution is needed given that findings from other designs (e.g. discordant siblings) are not consistent with a causal risk effect on mathematics ability.^13^^,^^14^

### Strengths and limitations

The current study utilized two large unselected epidemiological samples and was able to assess changes in the association between pre- and peri-natal risk factors and child outcomes across a 40-year period. The two cohorts included closely comparable measures of maternal smoking in pregnancy and a range of child outcomes.

There are also potential limitations. First, it is important to consider that consistent effects between cohorts do not rule out residual confounding. However, the current study employed a cross-cohort design to allow triangulation across different study designs to aid causal inference. Second, despite the outcome measures being similar, they were not identical. We used data from a calibration study to ensure comparability of the Rutter scale and the SDQ, and modelled the uncertainty of estimates for calibrated score.^34^ However, the use of Rubin’s rules in this context is conservative, meaning that estimates of levels of difficulties in the earlier cohort (NCDS) are less precise than for the later cohort (MCS). Second, maternal smoking and infant birth weight were assessed shortly after birth in the first cohort, but at age 9 months in the later cohort. There is also a possibility that mothers, particularly in the more recent cohort, under-reported smoking in pregnancy. However, evidence suggests that mothers do provide accurate information about smoking in pregnancy and child birth weight in comparison to medical records.^29^^,^^45–47^ Third, an important limitation is the lack of comparable information on smoking intensity and duration among mothers who smoked in pregnancy in the two cohorts. The information available suggested broadly similar patterns of smoking in the two cohorts, although there was some evidence of an increased numbers of heavy smokers in the later cohort (see Supplementary Table 3, available as Supplementary data at *IJE* online). We therefore cannot rule out the possibility that differences in patterns of association across the two cohorts reflect differences in exposure to maternal smoking during pregnancy. Fourth, though response rates were high in both cohorts, there was some missing data, which was more pronounced for children from more deprived family backgrounds. To address this problem, we used sample-specific attrition weights to correct for selective non-response. Other limitations include that maternal smoking and LBW were reported at the same time, increasing the possibility of a spurious association, and that measures of family adversity were assessed at a single time point only (age 7 years).

### Implications

Maternal smoking in pregnancy can indisputably cause harm both to mothers and the developing foetus. There are likely causal effects of maternal smoking in pregnancy on risk of a range of pregnancy complications, foetal mortality and still birth, LBW and prematurity.^43^ Public health education and availability of smoking-cessation interventions have helped reduce rates of smoking in general, and of maternal smoking in pregnancy in particular, with important benefits for maternal and child health. Nevertheless, smoking in pregnancy is still relatively common in many countries,^41^^,^^42^ and efforts to further reduce the harms caused by smoking are clearly an important population health priority.

Another priority is improving understanding of the causal or non-causal nature of associations linking early life risk exposures and child development more broadly. This understanding is essential if preventative interventions designed to improve specific child outcomes, such as child mental health, behaviour or academic attainment, are to be successful. A range of different study designs that go beyond standard multivariate adjustment are needed to draw robust conclusions. In the case of maternal smoking in pregnancy there is now substantial evidence that associations with maternal smoking and child conduct problems, hyperactivity/ADHD and cognitive attainment at least in part reflect the influence of shared confounding factors. If true, then efforts to reduce maternal smoking in pregnancy are unlikely to lead to population health improvements in these important aspects of children’s development.^48^ Identifying and testing alternative modifiable causal factors that could be targeted to improve these outcomes is an important research priority.

## Conclusions

The current study highlights the utility of the cross-cohort design for aiding causal inference in observational epidemiology. Triangulation of findings across different study designs is crucial for drawing reliable conclusions about the importance of putative risk factors in child development. The study adds to prior research that has highlighted likely causal effects of maternal smoking on child birth weight. In contrast, associations between maternal smoking and child conduct problems and hyperactivity differed markedly between the two cohorts, implicating the likely role of confounders.

## Funding

This work was supported by the Medical Research Council [grant number MR/J01348X/1] and by support to S.C. from the Waterloo Foundation. R.S. is supported by the Economic and Social Research Council grant award [ES/N003098/1]. A.P. is part funded by the National Institute for Health Research (NIHR) Biomedical Research Centre at South London and Maudsley NHS Foundation Trust and King’s College London. The views expressed are those of the author(s) and not necessarily those of the NHS, the NIHR or the Department of Health and Social Care.

## Conflict of interest

None declared.

## Supplementary Material

dyaa001_Supplementary_Data
