# Supplementary material for: Using a cross-cohort comparison design to test the role of maternal smoking in pregnancy in child mental health and learning: evidence from two UK cohorts born four decades apart
Source: Int J Epidemiol. 2020 Feb 10;49(2):390–9. doi: 10.1093/ije/dyaa001 (PMC7266557; doi:10.1093/ije/dyaa001)
Supplement: dyaa001_Supplementary_Data [file dyaa001_supplementary_data.docx]

**SUPPLEMENTARY MATERIAL**

**Supplementary table 1:** Cross-cohort comparison of associations between maternal smoking in pregnancy and child conduct/ hyperactivity problems in the abnormal range

|  |  |  | 1958 cohort (NCDS)  OR (95% CI), p | 2000/1 cohort (MCS)  OR (95% CI), p | cohort x smoking interaction  OR (95% CI), p |
| --- | --- | --- | --- | --- | --- |
| **Boys** | Conduct | Unadjusted | 1.25 (1.10, 1.1.42), p.001 | 2.40 (1.98, 2.91), p<.001 | 1.39 (1.34, 1.43), p<.001 |
|  |  | ^1^Adjusted model |  |  | 1.39 (1.28, 1.51), p<.001 |
|  | Hyperactivity | Unadjusted | 1.21 (1.08, 1.37), p.001 | 1.84 (1.56, 2.17), p<.001 | 1.23 (1.11, 1.36), p<.001 |
|  |  | ^1^Adjusted model |  |  | 1.13 (.95, 1.34), p.179 |
| **Girls** | Conduct | Unadjusted | 1.23 (1.09, 1.39), p.001 | 2.00 (1.62, 2.48), p<.001 | 1.28 (1.25, 1.30), p<.001 |
|  |  | ^1^Adjusted model |  |  | 1.20 (1.14, 1.26), p<.001 |
|  | Hyperactivity | Unadjusted | 1.21 (1.08, 1.36), p.001 | 1.61 (1.30, 1.98), p<.001 | 1.15 (1.02, 1.29), p.019 |
|  |  | ^1^Adjusted model |  |  | 1.13 (.91, 1.38), p.268 |

^1^Adjusting for home ownership; married at birth; Low maternal education; occupational status, maternal age at birth.

NCDS: National Child Development Study (1958 birth cohort); MCS: Millennium Cohort Study (2000/1 cohort)

**Supplementary table 2:** Cross-cohort comparison of associations between maternal smoking in pregnancy and child reading and maths in abnormal range

|  |  |  | 1958 cohort  (NCDS)  OR (95% CI), p | 2000/1 cohort  (MCS)  OR (95% CI), p | Cohort x smoking interaction  OR (95% CI), p |
| --- | --- | --- | --- | --- | --- |
| **Boys** | Reading | Unadjusted | 1.62 (1.40, 1.87), p<.001 | 2.40 (1.97, 2.92), p<.001 | 1.22 (1.08, 1.37), p.001 |
|  |  | ^1^Adjusted model |  |  | 0.98 (.82, 1.17), p.813 |
|  | Mathematics | Unadjusted | 1.66 (1.45, 1.90), p<.001 | 2.03 (1.66, 2.47), p<.001 | 1.10 (0.98, 1.25), p.106 |
|  |  | ^1^Adjusted model |  |  | 0.93 (.77, 1.13), p.471 |
| **Girls** | Reading | Unadjusted | 1.57 (1.36, 1.82), p<.001 | 2.08 (1.68, 2.58), p<.001 | 1.15 (1.01, 1.31), p.038 |
|  |  | ^1^Adjusted model |  |  | 0.89 (.72, 1.10), p.276 |
|  | Mathematics | Unadjusted | 1.55 (1.35, 2.14), p<.001 | 1.72 (1.39, 2.14), p<.001 | 1.06 (0.93, 1.20), p.407 |
|  |  | ^1^Adjusted model |  |  | 0.87 (.71, 1.96), p.175 |

^1^Examing interactions, adjusting for home ownership; married at birth; Low maternal education; occupational status, maternal age at birth

NCDS: National Child Development Study, 1958 birth cohort; MCS: Millennium Cohort Study (2000/1 cohort)

**Supplementary table 3:** Number of cigarettes smoked during pregnancy by mothers who reported smoking

|  | 1-4 daily | 5-9 daily | 10-14 daily | 15-19 daily | 20-24 daily | 25-29 daily | 30+ daily | variable |
| --- | --- | --- | --- | --- | --- | --- | --- | --- |
| NCDS | 20.4% | 26.4% | 24.9% | 5.6% | 4.2% | 0.7% | 0.7% | 17.1% |
| MCS | 27.2% | 28.8% | 22.3% | 7.9% | 9.9% | 1.0% | 2.8% | -- |

NCDS: National Child Development Study, 1958 birth cohort; MCS: Millennium Cohort Study (2000/1 cohort)

It was not possible to compare the intensity of smoking during pregnancy between NCDS and MCS. In NCDS 17.1% were classified as ‘variable’ smokers – these women would have smoked a variable number of cigarettes per day during pregnancy and therefore it was not possible to classify them (e.g., as medium or heavy smokers).
